# Socioeconomic determinants and inequalities in exclusive breastfeeding among children in Peru

**DOI:** 10.3389/fnut.2022.1073838

**Published:** 2022-12-15

**Authors:** Akram Hernández-Vásquez, Rodrigo Vargas-Fernández

**Affiliations:** ^1^Centro de Excelencia en Investigaciones Económicas y Sociales en Salud, Vicerrectorado de Investigación, Universidad San Ignacio de Loyola, Lima, Peru; ^2^Faculty of Health Sciences, Universidad Científica del Sur, Lima, Peru

**Keywords:** exclusive breastfeeding, socioeconomic factors, social inequalities, cross-sectional studies, Peru

## Abstract

**Introduction:**

Although Latin America and the Caribbean have one of the highest prevalences of exclusive breastfeeding (EBF), the countries in this region have socioeconomic determinants that influence the frequency of this practice and do not allow achieving the 70% target recommended by the World Health Organization. Therefore, the objective of the study was to examine the socioeconomic determinants and perform a decomposition analysis of socioeconomic inequalities in EBF in Peruvian children 6 to 59 months of age.

**Methods:**

A cross-sectional study was carried out using the 2021 Demographic and Family Health Survey. The dependent variable for the study was EBF up to 6 months of age and the wealth index variable was used to perform the inequality analysis. Poisson log generalized linear regression models were fitted to evaluate the association between EBF and the independent variables, and concentration curves and Erreygers concentration index decomposition were used to analyze inequalities in EBF.

**Results:**

A total of 9926 surveyed participants were included. The prevalence of EBF was 70.5% (95% confidence interval: 69.2-71.8). Women who were married, self-identified as native, received EBF training, resided in the highlands and jungle, and their child was the second or older showed a higher likelihood of EBF. In the inequality analysis, EBF was concentrated among the poorest mothers and the major contributors were residing in the highlands and jungle and belonging to the middle and wealthy quintiles.

**Discussion:**

Our findings suggest that the main strategies to encourage the practice of EBF should be focused on all mothers regardless of their socioeconomic status in order to reduce the EBF gap between richer and poorer women.

## 1 Introduction

Breastfeeding is one of the public health strategies that provides important benefits for maternal and infant health (1). Human milk is composed of water, lipids (long-chain polyunsaturated acids), carbohydrates (oligosaccharides), low concentrations of proteins, minerals and vitamins, as well as growth and immunological factors (2, 3). These components reduce infant morbidity and mortality from infectious diseases (infant diarrhea and acute respiratory infection), while in breastfeeding mothers they prevent the onset of chronic diseases (breast cancer, ovarian cancer, diabetes, among others) and prolong the inter-gestational period of births (1, 4). Although the World Health Organization (WHO) and the United Nations Children’s Fund (UNICEF) promote exclusive breastfeeding (EBF) for the first 6 months of life and continuing this practice up to 24 months of age (5), it is estimated that less than 50% of infants under 6 months of age have received EBF in the world. (6). In addition, the absence of EBF in children is reported to have generated more than 13 million disability-adjusted life years and more than 140 thousand deaths in 2019 (7). These indicators are reflected in higher health spending for healthcare systems due to the burden of disease that may be preventable with EBF (8, 9).

Low- and middle-income countries (LMICs) have a higher prevalence and duration of EBF compared to high-income countries. However, LMICs have EBF figures (38.7% in 2018) that are well below the figure recommended (70%) by the WHO for 2030, and have a higher number of child deaths attributed to the absence of EBF (4, 7, 10). More than 30 million children in LMICs do not receive EBF due to socioeconomic, cultural and individual determinants that affect EBF practice decisions and behaviors (10). These determinants are related to the woman’s age, maternal education, maternal employment, maternal nutritional status, number of antenatal care visits, place and route of delivery, newborn characteristics, social influence and traditional practices that affect the onset and duration of EBF (11–13), which vary between and within countries, accentuating health inequalities (8). These inequalities are present in most countries, where poorer and less educated mothers have a higher frequency and duration of EBF than their counterparts (1). In this sense, since EBF is one of the most cost-effective practices in health, the strategies that promote EBF are oriented to all women to reduce the negative effects on child health indicators; however, there are socioeconomic inequalities that influence EBF practices and generate an unequal distribution of this practice among women (14, 15).

Latin America and the Caribbean (LAC) is one of the regions made up of LMIC countries, in which the prevalence of EBF has increased from 37.3% in 2000 to 51.7% in 2019, being one of the regions with the highest prevalence of EBF (16). However, the frequency of EBF decreases the older the age of the child (67.6% in children aged 0 months vs. 22.2% in children aged 5 months), reflecting a shorter duration of EBF in this region (17). Within the LAC countries, socioeconomic determinants influencing EBF practices have been observed, with a high educational and economic level of the mother being positively associated with the frequency and duration of EBF (18, 19). In Peru, the Demographic and Family Health Survey (ENDES–acronym in Spanish) shows that EBF increased from 65.2% in 2015 to 68.4% in 2020 (20). However, a pattern similar to that of other LAC countries is observed, in which the frequency of EBF decreases the older the child is (74% in 1-month-old children vs. 35% in 6-month-old children) reflecting a limited duration of EBF (21). Peru is an unequal and heterogeneous country, presenting socioeconomic characteristics that differ from one person to another, and predispose the emergence of inequalities in various health areas, such as EBF practices. Thus, the assessment of socioeconomic inequalities would help to define, describe and understand the nature of this problem in the Peruvian territory in order to develop strategies and policies to address these inequalities.

The information obtained from the Peruvian population in the year 2021 would help to understand the possible changes that have occurred in EBF practices. Likewise, this updated information would allow identifying the progress that has been made toward the achievement (70%) of EBF by 2030, especially considering that the LAC region presented a figure much lower than expected for that year. Therefore, the objective of the present study was to examine the socioeconomic determinants and perform a decomposition analysis of socioeconomic inequalities in EBF in Peruvian children aged 6 to 59 months using the 2021 ENDES.

## 2 Materials and methods

### 2.1 Data

This was a cross-sectional study developed with data from the 2021 ENDES (22). The objective of the 2021 ENDES was to provide updated information on demographic dynamics, the health status of mothers and children under 5 years of age, information on the status and factors associated with non-communicable and communicable diseases, as well as access to diagnostic and treatment services. This information allows estimating the indicators used in the monitoring, evaluation and formulation of population and family health programs in the country (23).

The 2021 ENDES is a population-based survey conducted between January and December 2021 by the National Institute of Statistics and Informatics (INEI–acronym in Spanish). The target population is private households and their members, persons who are usual residents and those who, not being residents, stayed overnight in the dwelling the night before the day of the interview, including all women from 12 to 49 years of age and children under 5 years of age, one person from 15 years of age or older per private household, and all children under 12 years of age. For the selection of the sample, the sampling framework was constituted by the statistical and cartographic information from the XII National Population Census and VII National Housing Census of 2017 (CPV 2017), and the cartographic material updated for this purpose in the cartographic updating process carried out for the execution of the ENDES. The sample is characterized as two-stage, probabilistic, balanced, stratified and independent, at the departmental level, by urban and rural area (23).

### 2.2 Sampling and data collection

The sampling units in the urban area were conglomerate and the private dwellings and were the rural census area and private dwellings in the rural area. The research unit of the survey was made up of the usual residents of private dwellings in urban and rural areas of the country who have spent the night before the survey in the selected dwelling. The collection of coverage information in the selected dwellings was carried out using a mobile device: Tablet. The method used was by direct interview (face-to-face) and telephone interview, conducted by personnel duly trained for the collection of this information. Further details on the sampling process, design and contents of the 2021 ENDES can be found in the annual report and technical data sheet (22, 23).

### 2.3 Inclusion/exclusion criteria

Children aged 6 to 59 months who were alive at the time of the survey were included. We also included information on the mothers between 15 and 49 years of age. If the woman had two or more children, the most recent child was included in the study in order to have pre- and perinatal information, given that the 2021 ENDES only collects this information for the last newborn (22). Children with missing data on the variables of interest were excluded.

### 2.4 Measures

The dependent variable for the study was EBF until 6 months of age. The variable was created from the question: During the first 6 months of life, did (Name of child) receive only breast milk without including other foods or liquids? This question was categorized and coded as Yes (1) and No (0).

The mother’s age group (15–19, 20–34, 35–49 years), educational level (up to primary, secondary, higher), marital status (single/widowed/divorced, married, cohabiting), ethnic self-identification (non-native, native), child’s birth order (0–1, 2–3, 4 or more), sex of child (female, male), place of delivery (home, health center), mode of delivery (vaginal, cesarean), antenatal care visits (0–7, 8 or more), breastfeeding training (no, yes), wealth quintile of household (poorest, poorer, middle, richer, richest), area of residence (rural, urban), and region of residence (coast, highlands, jungle) were independent variables included in the analysis of associated factors. Likewise, for the analysis of inequalities, the wealth index of the household was used as an independent variable and as a continuous variable (24). These variables were selected according to similar studies available on the subject (25–31).

### 2.5 Statistical analysis

Sampling weights were applied in all our analyses to adjust for unequal cluster sizes, stratifications and to ensure that our findings adequately represent the national/regional representation of the survey results. Details of the design and sampling weights can be found in the 2021 ENDES data sheet (23). Data analysis included descriptive, inferential and inequality analysis. Descriptive analysis was used to report the frequency distribution of the study variables. Descriptive analysis included a presentation of frequency tables and figures. Chi-square tests were performed to determine differences between the proportions of independent and dependent variables. Poisson log generalized linear regression models (crude and adjusted) were fitted to evaluate the association between EBF and independent variables in the inferential analysis. Potential factors were selected when the variables obtained a *p*-value < 0.20 in the bivariate model. For each regression, the measures used to assess the association between the dependent and independent variables were the crude (PR) and adjusted prevalence ratio (aPR) and the 95% confidence interval (CI). Multicollinearity among the independent variables was tested using the “collin” command and the results revealed no evidence of multicollinearity (mean 1.34, maximum 2.16 and minimum 1.00).

Concentration curves (CC) and the Erreygers concentration index decomposition were used to analyze socioeconomic and territorial inequalities in EBF (32–34). CC stratified according to the available variables following the acronym PROGRESS (35) were used to plot the cumulative percentage of the EBF on the *y*-axis vs. the cumulative percentage of the population according to the wealth index as a socioeconomic indicator starting on the *x*-axis with those with the lowest wealth index, where the curve above/below the equality line indicates that EBF is concentrated in the population with the lowest/highest wealth index (34). Similarly, concentration indices measure the magnitude of inequality, where if the concentration index takes a negative value the concentration of EBF is among the poorest, and on the contrary, if the concentration index takes a positive value the concentration is among the richest (34). In the absence of socioeconomic inequality, the concentration curve is located on the diagonal line (equality line) and the concentration index is zero. Finally, the Erreygers concentration index was decomposed based on a generalized linear model binomial distribution and identity link (36) and following the methodology described by O’Donnell et al. (34) to quantify the contribution of the variables included to inequality in EBF. The concentration index decomposition reports elasticity, concentration, the contribution, and the percentage of contribution to the inequality for each independent variable (34, 37). The elasticity denotes the change in the outcome of interest associated with a one-unit change in the independent variable. The concentration index represents the concentration index of the independent variables with reference to the wealth index (34, 37). The contribution and percentage contribution represents the absolute and relative contribution of each independent variable included in the model to the overall socioeconomic-related inequality in the outcome of interest. A positive or negative value in the contribution or percentage contribution results in an increase or decrease in the inequality (34, 37).

Statistical significance was set at 5%. All statistical analyses and CC were performed in Stata 17 (StataCorp, College Station, TX, USA). We used the ggplot2 package in R (R V.3.4.1 and RStudio V.1.3.959) to perform the inequality plots according to mother’s educational level and area of residence.

## 3 Results

### 3.1 Population characteristics

A total of 9,926 women aged 15 to 49 years with their last child aged 6 to 59 months were included in the analysis. Among the sociodemographic characteristics, it was observed that the greatest proportion of the women belonged to the 20–34 years of age group (66.1%), had a secondary education (49.5%), resided in an urban area (68.6%) and belonged to the poorest wealth quintile (31.7%), while the majority of their children were male (50.5%), and were their second or third child (52.4%). It was also found that the majority had 8 or more antenatal care visits (67.4%), their pregnancy ended vaginally (80.4%), and was carried out in a health facility (92.4%), while 28.2% of women did not receive training on breastfeeding practices. The characteristics of both the women and their children are shown in Table 1.

**TABLE 1:** Characteristics of the Peruvian women aged 15–49 years and their children aged 6–59 months included in the study (*n* = 9,926).

Characteristics	*n*	%*
**Maternal age groups (years)**		
15–19	451	4.5
20–34	6572	66.1
35–49	2903	29.3
**Educational level**		
Up to primary	2309	21.8
Secondary	5048	49.5
Higher	2569	28.8
**Marital status**		
Single/widowed/divorced	1671	18.2
Married	1846	18.7
Cohabiting	6409	63.1
**Ethnic self-identification**		
Non-native	6094	70.1
Native	3832	29.9
**Order of birth**		
0–1	2672	27.8
2–3	5136	52.4
4 or more	2118	19.8
**Sex of child**		
Female	4950	49.5
Male	4976	50.5
**Place of delivery**		
Home	712	7.6
Health center	9214	92.4
**Antenatal care visits**		
0–7	3032	32.6
8 or more	6894	67.4
**Mode of delivery**		
Vaginal	8124	80.4
Cesarean	1802	19.6
**Breastfeeding training**		
No	2715	28.2
Yes	7211	71.8
**Wealth index**		
Poorest	3826	31.7
Poorer	2734	25.3
Middle	1764	20.4
Richer	1043	13.5
Richest	559	9.2
**Area of residence**		
Rural	3886	31.4
Urban	6040	68.6
**Region of residence**		
Coast	3138	46.2
Highlands	3851	31.4
Jungle	2937	22.4

*Estimates include the weights and ENDES 2021 sample specifications. Percentages may not total 100 due to rounding.

### 3.2 Prevalence of EBF according to sociodemographic characteristics

**FIGURE 1:**
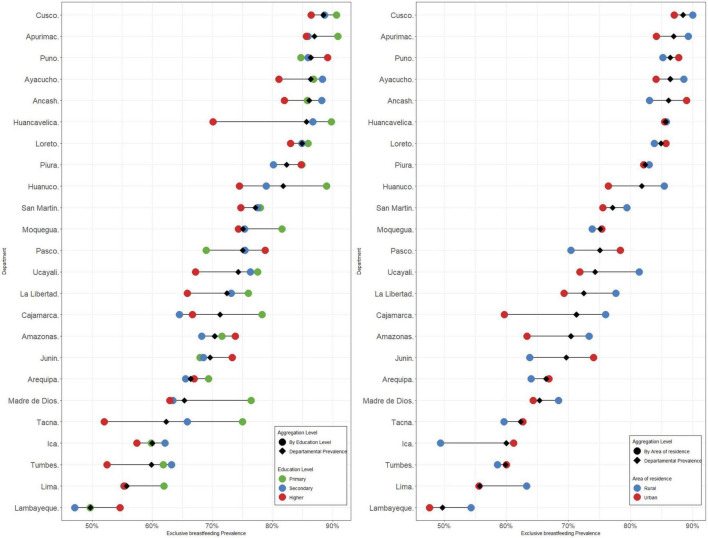
Prevalence of exclusive breastfeeding (EBF) by department and socioeconomic characteristics.

The prevalence of EBF was 70.5% in the study population. The women with the highest proportion of EBF had no or only primary education (78.2%), were married (72.5%), self-identified as natives (77.3%), resided in a rural area (78.6%) and in the highlands (80.1%), and belonged to the poorest quintiles (Q1 [79.6%] and Q2 [72.7%]), while the children with the highest proportions of EBF were the fourth or oldest child in birth order (76.8%). Likewise, the highest prevalence of EBF was found in women whose pregnancy was delivered vaginally (71.5%) and in a home (76.4%), and in those who received training on breastfeeding practices (71.7%) (Table 2). Regarding departmental prevalences (the 24 departments of Peru were included, with the Constitutional Province of Callao being considered within the department of Lima), the departments of Arequipa, Lima, Ica, Junín, Lambayeque, Madre de Dios, Tacna, and Tumbes had EBF figures below the 70% recommended by the WHO (Figure 1). Likewise, regarding departmental prevalences according to sociodemographic characteristics such as area of residence and educational level, it was observed that the highest prevalences of EBF were found in rural areas and in children of mothers with no education or primary education (Figure 1).

**TABLE 2:** Prevalence of exclusive breastfeeding (EBF) by characteristics of the Peruvian women aged 15–49 years and their children aged 6–59 months included in the study.

	Exclusive breastfeeding		
	
	No (*n* = 2,626)	Yes (*n* = 7,300)	
	
Characteristics	% (95% CI)	% (95% CI)	*P*-value*
Overall	29.5 (28.2-30.8)	70.5 (69.2-71.8)	
**Maternal age groups (years)**			
15–19	35.2 (29.8-41.0)	64.8 (59.0-70.2)	0.116
20–34	29.4 (27.8-31.0)	70.6 (69.0-72.2)	
35–49	28.9 (26.7-31.3)	71.1 (68.7-73.3)	
**Educational level**			
Up to primary	21.8 (19.6-24.1)	78.2 (75.9-80.4)	<0.001
Secondary	30.1 (28.4-31.8)	69.9 (68.2-71.6)	
Higher	34.4 (31.8-37.2)	65.6 (62.8-68.2)	
**Marital status**			
Single/widowed/divorced	34.6 (31.1-38.3)	65.4 (61.7-68.9)	0.002
Married	27.5 (24.8-30.4)	72.5 (69.6-75.2)	
Cohabiting	28.6 (27.1-30.2)	71.4 (69.8-72.9)	
**Ethnic self-identification**			
Non-native	32.4 (30.8-34.1)	67.6 (65.9-69.2)	<0.001
Native	22.7 (20.8-24.7)	77.3 (75.3-79.2)	
**Order of birth**			
0–1	34.5 (32.2-36.9)	65.5 (63.1-67.8)	<0.001
2–3	29.2 (27.4-31.0)	70.8 (69.0-72.6)	
4 or more	23.2 (20.9-25.7)	76.8 (74.3-79.1)	
**Sex of child**			
Female	28.7 (27.0-30.4)	71.3 (69.6-73.0)	0.188
Male	30.3 (28.5-32.2)	69.7 (67.8-71.5)	
**Place of delivery**			
Home	23.6 (20.2-27.4)	76.4 (72.6-79.8)	0.002
Health center	30.0 (28.6-31.4)	70.0 (68.6-71.4)	
**Antenatal care visits**			
0–7	30.5 (28.1-32.9)	69.5 (67.1-71.9)	0.326
8 or more	29.0 (27.6-30.6)	71.0 (69.4-72.4)	
**Mode of delivery**			
Vaginal	28.5 (27.1-29.9)	71.5 (70.1-72.9)	0.001
Cesarean	33.8 (30.9-36.8)	66.2 (63.2-69.1)	
**Breastfeeding training**			
No	32.6 (30.3-35.0)	67.4 (65.0-69.7)	0.002
Yes	28.3 (26.8-29.8)	71.7 (70.2-73.2)	
**Wealth index**			
Poorest	20.4 (18.8-22.2)	79.6 (77.8-81.2)	<0.001
Poorer	27.3 (25.0-29.7)	72.7 (70.3-75.0)	
Middle	34.2 (30.8-37.7)	65.8 (62.3-69.2)	
Richer	38.9 (35.2-42.6)	61.1 (57.4-64.8)	
Richest	42.8 (37.7-48.0)	57.2 (52.0-62.3)	
**Area of residence**			
Rural	21.4 (19.7-23.3)	78.6 (76.7-80.3)	<0.001
Urban	33.2 (31.6-34.9)	66.8 (65.1-68.4)	
**Region of residence**			
Coast	39.1 (36.8-41.5)	60.9 (58.5-63.2)	<0.001
Highlands	20.0 (18.4-21.8)	80.0 (78.2-81.6)	
Jungle	22.9 (21.0-25.0)	77.1 (75.0-79.0)	

Estimates include the weights and ENDES 2021 sample specifications. The proportions are distributed by rows. *The *p*-value was calculated using the Rao-Scott Chi-squared test. CI, confidence interval.

### 3.3 Factors associated with EBF

In relation to the factors associated with EBF, in the crude model all the variables included in the study, with the exception of the number of antenatal care visits, were associated with the practice of EBF in children aged 6–59 months. In the adjusted model, women who were married, self-identified as native, received training on breastfeeding practices, resided in the highlands and jungle regions, and their child was second or older in birth order were more likely to follow EBF practices with their children aged 6–59 months, while in the middle, richer and richest quintiles, the probability of performing EBF decreased (Table 3).

**TABLE 3:** Factors associated with exclusive breastfeeding (EBF) among infants between 6 and 59 months of age.

Characteristics	Crude model		Adjusted model*	
		
	PR (95% CI)	*P*-value	aPR (95% CI)	*P*-value
**Maternal age groups (years)**				
15–19	Reference		Reference	
20–34	1.09 (1.00-1.19)	0.058	1.05 (0.96-1.15)	0.289
35–49	1.10 (1.00-1.20)	0.048	1.02 (0.92-1.13)	0.718
**Educational level**				
Up to primary	Reference		Reference	
Secondary	0.89 (0.86-0.93)	<0.001	0.98 (0.94-1.02)	0.371
Higher	0.84 (0.80-0.88)	<0.001	1.00 (0.94-1.07)	0.951
**Marital status**				
Single/widowed/divorced	Reference		Reference	
Married	1.11 (1.04-1.19)	0.002	1.08 (1.01-1.16)	0.023
Cohabiting	1.09 (1.03-1.16)	0.004	1.05 (0.99-1.11)	0.107
**Ethnic self-identification**				
Non-native	Reference		Reference	
Native	1.14 (1.10-1.18)	<0.001	1.05 (1.01-1.09)	0.022
**Order of birth**				
0–1	Reference		Reference	
2–3	1.08 (1.03-1.13)	<0.001	1.07 (1.02-1.12)	0.009
4 or more	1.17 (1.12-1.23)	<0.001	1.11 (1.04-1.19)	0.001
**Sex of child**				
Female	Reference		Reference	
Male	0.98 (0.94-1.01)	0.189	0.98 (0.94-1.01)	0.172
**Place of delivery**				
Home	Reference		Reference	
Health center	0.92 (0.87-0.96)	0.001	1.05 (1.00-1.11)	0.067
**Antenatal care visits**				
0–7	Reference		No included	
8 or more	1.02 (0.98-1.06)	0.331		
**Mode of delivery**				
Vaginal	Reference		Reference	
Cesarean	0.93 (0.88-0.97)	0.002	0.98 (0.94-1.03)	0.475
**Breastfeeding training**				
No	Reference		Reference	
Yes	1.06 (1.02-1.11)	0.002	1.04 (1.00-1.09)	0.032
**Wealth index**				
Poorest	Reference		Reference	
Poorer	0.91 (0.88-0.95)	<0.001	0.96 (0.91-1.00)	0.077
Middle	0.83 (0.78-0.88)	<0.001	0.91 (0.85-0.97)	0.005
Richer	0.77 (0.72-0.82)	<0.001	0.87 (0.80-0.94)	0.001
Richest	0.72 (0.65-0.79)	<0.001	0.83 (0.74-0.92)	0.001
**Area of residence**				
Rural	Reference		Reference	
Urban	0.85 (0.82-0.88)	<0.001	1.03 (0.98-1.07)	0.277
**Region of residence**				
Coast	Reference		Reference	
Highlands	1.31 (1.26-1.37)	<0.001	1.22 (1.16-1.28)	<0.001
Jungle	1.27 (1.21-1.33)	<0.001	1.18 (1.13-1.24)	<0.001

Estimates include the weights and ENDES 2021 sample specifications. *Adjusted for the variables shown in the column. PR, prevalence ratio; aPR, adjusted prevalence ratio; CI, confidence interval.

### 3.4 Inequality in the distribution of EBF: Concentration curves

**FIGURE 2:**
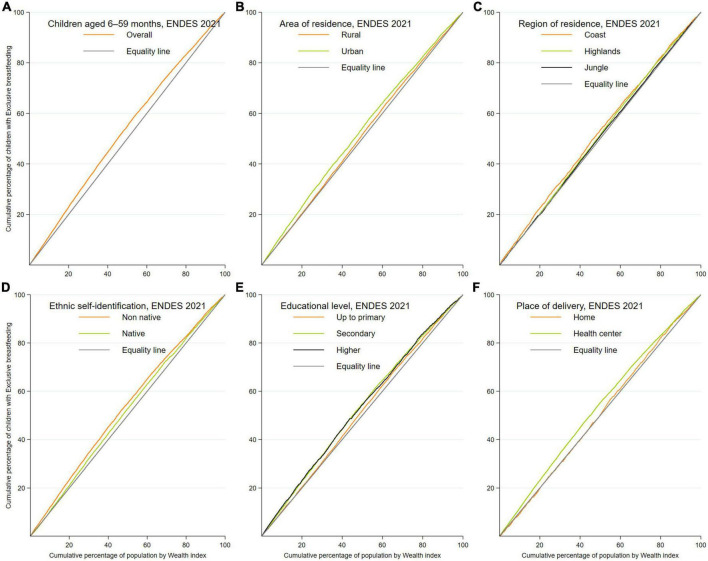
Concentration curves (CC) of exclusive breastfeeding (EBF) among infants between 6 and 59 months of age. **(A–F)** Concentration curves according to the sociodemographic characteristics of the population included.

The CC for EBF practice are shown in Figure 2. Overall, EBF in children aged 6–59 months was concentrated among poorer women. In addition, the pro-poor concentration was found to be more marked in women who resided in an urban area, self-identified as non-native, had secondary or higher education, and their last delivery was attended in a health facility. On the other hand, the CCs of the natural region categories (coast, highlands, and jungle) were close to the line of equality, indicating less EBF inequality.

### 3.5 Decomposition analysis of the inequality in EBF distribution

The decomposition analysis is shown in Table 4. Overall, it was found that women aged 20–34 years (−0.0014), who had a secondary education (−0.0665), were cohabiting (−0.1330), self-identified as native (−0.2268), resided in the highlands (−0.3820) and jungle (−0.2680) regions, belonged to the poor wealth quintile (−0.1147), had received training on breastfeeding practices (−0.0642) and whose children were fourth or higher in birth order (−0.2079) presented a negative value in the concentration index, which indicates that they were concentrated in the poorer wealth quintiles. On the other hand, the results of the inequality decomposition analysis showed that the main contributors for explaining the economic inequality gap between the poor and rich in terms of EBF were residing in the highlands (13.0%) and jungle (5.3%) regions and belonging to the middle (3.2%) and rich (3.8%) wealth quintiles.

**TABLE 4:** Decomposition of concentration indices of exclusive breastfeeding (EBF) among infants between 6 and 59 months of age.

Characteristics	Elasticity	Concentration index	Contribution	% Contribution
**Maternal age groups (years)**				
15–19	Reference	Reference	Reference	Reference
20–34	0.0225	–0.0014	0.0000	0.0
35–49	0.0007	0.0422	0.0000	0.0
**Educational level**				
Up to primary	Reference	Reference	Reference	Reference
Secondary	–0.0096	–0.0665	0.0006	–0.4
Higher	0.0004	0.4871	0.0002	–0.1
**Marital status**				
Single/widowed/divorced	Reference	Reference	Reference	Reference
Married	0.0119	0.0823	0.0010	–0.5
Cohabiting	0.0226	–0.1330	–0.0030	1.7
**Ethnic self-identification**				
Non-native	Reference	Reference	Reference	Reference
Native	0.0136	–0.2268	–0.0031	1.7
**Order of birth**				
0–1	Reference	Reference	Reference	Reference
2–3	0.0378	0.1054	0.0040	–2.2
4 or more	0.0214	–0.2079	–0.0044	2.5
**Sex of child**				
Female	Reference	Reference	Reference	Reference
Male	–0.0104	0.0100	–0.0001	0.1
**Place of delivery**				
Home	Reference	Reference	Reference	Reference
Health center	0.0521	0.2096	0.0109	–6.0
**Antenatal care visits**				
0–7	Reference	Reference	Reference	Reference
8 or more	0.0027	0.0485	0.0001	–0.1
**Mode of delivery**				
Vaginal	Reference	Reference	Reference	Reference
Cesarean	–0.0039	0.1861	–0.0007	0.4
**Breastfeeding training**				
No	Reference	Reference	Reference	Reference
Yes	0.0334	–0.0642	–0.0021	1.2
**Wealth index**				
Poorest	Reference	Reference	Reference	Reference
Poorer	–0.0119	–0.1147	0.0014	–0.8
Middle	–0.0209	0.2801	–0.0059	3.2
Richer	–0.0189	0.3675	–0.0069	3.8
Richest	–0.0159	0.3331	–0.0053	2.9
**Area of residence**				
Rural	Reference	Reference	Reference	Reference
Urban	0.0177	0.7580	0.0134	–7.4
**Region of residence**				
Coast	Reference	Reference	Reference	Reference
Highlands	0.0619	–0.3820	–0.0236	13.0
Jungle	0.0356	–0.268	–0.0096	5.3
Residual −0.1482				

Estimates include the weights and ENDES 2021 sample specifications. %, percentage.

## 4 Discussion

The present study sought to determine the associated factors and quantify the contribution of contextual and compositional factors on socioeconomic inequalities in EBF practices in Peruvian children aged 6–59 months. It was found that 7 out of 10 Peruvian children aged 6 to 59 months received EBF, with children of mothers with no or a primary education, residing in a rural area and in the departments that make up the highlands and jungle having the highest prevalences of EBF. Regarding the factors associated with EBF, several maternal, child and household characteristics were found to increase the probability of EBF in their children aged 6 to 59 months. In terms of the inequality analysis, the practice of EBF was concentrated among the poorest women, while the main contributors in the decomposition analysis were the natural region of residence and the wealth index.

The prevalence of EBF in Peruvian children aged 6 to 59 months was 70.5%. This finding is higher than that reported in studies estimating the prevalence of EBF in LMIC (38.7%) (10), and high-income countries (18%) (38). Likewise, this result is higher than that reported in LAC, in which the prevalence was estimated at 51.7% in 2019 (16). In this region, the countries with the highest prevalence of EBF were Bolivia (60.4%), Guatemala (53.2%), and Haiti (39.9%), while the countries with the lowest prevalence were the Dominican Republic (4.7%), Guyana (23.3%), and Honduras (23.8%) (16). A previous study that estimated the prevalence of EBF in Peru showed an increase from 63.6% in 2000 to 69.2% in 2018 (10), indicating that EBF figures continue to increase over the years, reaching 70.5% in 2021. It should be noted that by 2021, Peru had reached the figure of 70% of EBF recommended by the WHO for 2030. However, this prevalence is dissimilar within the country because the highest prevalences were found in women with a low educational level, residing in rural areas and the departments that have a greater distribution in the highlands and jungle of Peru. These findings could be attributed to various cultural and sociodemographic factors observed in the Peruvian territory. It has been described that cultural beliefs and practices on infant feeding could be a determining factor in the inclusion of breastfeeding, especially in regions of the highlands and jungle, where cultural traditions persist over time (39, 40). In addition, a high level of education in LMIC women could be associated with higher labor participation which would generate poor breastfeeding practices (41–43). Therefore, governmental institutions should carry out strategies for working mothers aimed at the inclusion of breastfeeding in the workplace. In addition, orientation programs on EBF are required in regions such as the departments that have a greater distribution in the Coast region, where the prevalence of EBF is low.

It was found that married women, who self-identified as natives, received training on breastfeeding practices, resided in the highlands and jungle regions, and their child was the second or older in birth order were more likely to have perform EBF. These results are similar to those reported in studies conducted in Ethiopia (29), Iran (25), Kenya (27), and Malawi (28), in which women who were married, belonged to specific ethnic groups, received breastfeeding counseling, had a low economic income, and the child was third or fourth in birth order were associated with a higher likelihood of EBF. These findings could be explained by multiple cultural, socioeconomic, and demographic determinants that influence breastfeeding practices. Regarding the influence of marital status on EBF, the biomedical literature indicates that the social environment of married women affects postpartum health and behavior (44, 45), increasing the likelihood of EBF, and even the husband’s attitudes and beliefs may be relevant when making decisions about infant feeding because breastfeeding is strongly influenced by social and cultural aspects of the woman’s environment (46–48). Likewise, Peruvian women who self-identify as native guide breastfeeding practices based on culture-specific beliefs, with which mothers’ concepts about the baby’s development influence infant feeding. In addition, there are some natural resources (animals, plants, and minerals) in the Andean region of Peru that have nutritional properties and are implemented in the diet of breastfeeding mothers as a belief of increased production of breast milk (39, 40).

Several studies have reported that receiving counseling on breastfeeding practices during the prenatal and postnatal period increases the likelihood that offspring will receive EBF (49). In fact, breastfeeding counseling is a WHO global recommendation to increase breastfeeding initiation rates and ensure EBF because this intervention strengthens individual interactions between health workers and mothers (either face-to-face or by telephone), and enables mothers to make appropriate infant feeding decisions (49). These interactions allow counselors to respond in a timely manner to challenges the mother may face during breastfeeding to ensure adequate EBF (49). In relation to birth order, in contrast to our finding, the biomedical literature mentions that the higher the birth order, the less likely mothers are to breastfeed their children, with the fourth child or higher in the order being approximately 20% less likely to be breastfed compared to the first (50). However, our finding could be attributed to the fact that mothers who have older children have higher levels of confidence about breastfeeding and perceive less problematic feeding behavior than first-time mothers (51, 52). In this sense, the creation of strategies that promote EBF should ensure a safe family environment, include indigenous mothers in educational programs while respecting their cultural traditions, and prioritize breastfeeding counseling in the prenatal and postnatal periods.

Based on the CC evaluated in the present study, it was found that EBF was concentrated among women with a poorer socioeconomic status. This finding is different from that described in studies conducted in Bangladesh, Nigeria, Norway, and the United States (11, 12, 53, 54), which reported that EBF was concentrated in women with a higher economic income. In accordance with the biomedical literature, there is a positive correlation between formal education and socioeconomic status (55). In LMIC, women with low levels of formal education are observed to have higher prevalences than their counterparts, which is related to a decrease in the inequality gap in nutrition and health between richer and poorer children (56). However, women who are richer in LMIC face challenges related to infant nutrition, with increased marketing and advertising of breast milk substitutes in health systems and other media having led to a decrease in EBF, especially in terms of the affordability of these products compared to women who are richer in LMIC (57, 58). Likewise, the decomposition analysis showed that the main contributors to the inequality gap between poorer and richer women were residing in the highland and jungle regions and belonging to the middle and rich wealth quintiles. According to the literature, most Peruvian women living in the highlands and jungle regions have low socioeconomic levels, which could be associated with low exposure to advertising and marketing of breast milk substitutes and where cultural practices increase EBF (26). In this sense, the existing inequality in EBF in Peruvian women is in agreement with that reported in LMIC and persisted even during the pandemic. In this regard, WHO considers that a key component of infant care is to ensure equitable access to breast milk for all infants regardless of the socioeconomic status of the mothers (59). Particularly in Peru, cultural practices should be considered as playing a relevant role in these inequalities and should be addressed when considering health policies.

Our findings have implications for public health. First, although the prevalence of EBF in Peru has reached the 70% recommended by the WHO for the year 2030, there are socioeconomic inequalities that define the higher prevalence of EBF in poorer women. This problem should be addressed through the promotion of EBF in all socioeconomic strata to ensure EBF in all Peruvian children and that the increase in prevalence is constant. Second, guidelines that ensure EBF should include the cultural traditions and language of indigenous women residing in the highlands and jungle of Peru in order to provide a multicultural approach to improve counseling on breastfeeding practices. Third, breastfeeding protection policies in the workplace should be a priority for government institutions, because these interventions promote and ensure EBF through individual, interpersonal and organizational interaction. Fourth, policies should be created to minimize the advertising and marketing of breast milk substitutes, especially among women who are able and unconstrained to breastfeed their infants. Finally, strategies that promote EBF should be in line with the World Breastfeeding Collective, which seeks to ensure breastfeeding in a period of crisis such as the current COVID-19 pandemic (60).

The present study has some limitations. Due to its cross-sectional nature, causality cannot be established between the study variables due to a lack of temporality in their measurement. In addition, there could be recall bias due to some data coming from events that have occurred in the past, as well as inaccuracy during data collection by the interviewer. By using secondary databases, some confounding variables that could have relevance when exploring EBF could have been omitted, such as women’s cultural practices, women’s nutritional status, father’s education, knowledge about breastfeeding, whether the child received oral rehydration salts, drops and syrups, social support from institutions or other variables related to COVID-19. Despite these limitations, this study is based on the analysis of a survey that is representative at the national level, by department and by urban and rural area, and its execution contains standardized procedures carried out by duly trained personnel, which ensure the quality and measurement of the information collected.

In conclusion, the prevalence of EBF in children aged 6–59 months was found to be 70.5%, being an adequate prevalence for the 70% recommended by the WHO for 2030. However, there are departments in Peru that have not reached this prevalence. Women who are natives, who live in the highlands and jungle, who received counseling on EBF and whose child was the second or older were more likely to perform EBF. Likewise, the CC showed that the EBF was concentrated among children whose mothers were poorer, while the inequality decomposition analysis found that the main contributors to inequality resided in the highland and jungle regions and belonged to the middle and rich wealth quintiles. In this sense, current strategies that promote EBF should prioritize the inclusion of the traditional practices of women, the protection of EBF in the workplace, the promotion of breastfeeding practices without considering the socioeconomic level of the woman and the regulation of inadequate commercialization of breast milk substitutes to achieve a similar percentage of EBF in all departments of Peru.

## Data availability statement

Publicly available datasets were analyzed in this study. This data can be found here: http://iinei.inei.gob.pe/microdatos/.

## Author contributions

AH-V: conceptualization, data curation, formal analysis, investigation, project administration, software, and supervision. AH-V and RV-F: methodology, validation, visualization, and writing – original draft and review and editing. Both authors contributed to the article and approved the submitted version.
